# Socioeconomic status, white matter, and executive function in children

**DOI:** 10.1002/brb3.531

**Published:** 2016-08-02

**Authors:** Alexandra Ursache, Kimberly G. Noble, the Pediatric Imaging, Neurocognition and Genetics Study

**Affiliations:** ^1^ Sergievsky CenterColumbia UniversityNew York CityNYUSA; ^2^ Teachers CollegeColumbia UniversityNew York CityNYUSA

**Keywords:** adolescents, children, education, executive function, income, white matter

## Abstract

**Background:**

A growing body of evidence links socioeconomic status (SES) to children's brain structure. Few studies, however, have specifically investigated relations of SES to white matter structure. Further, although several studies have demonstrated that family SES is related to development of brain areas that support executive functions (EF), less is known about the role that white matter structure plays in the relation of SES to EF. One possibility is that white matter differences may partially explain SES disparities in EF (i.e., a mediating relationship). Alternatively, SES may differentially shape brain‐behavior relations such that the relation of white matter structure to EF may differ as a function of SES (i.e., a moderating relationship).

**Method:**

In a diverse sample of 1082 children and adolescents aged 3–21 years, we examined socioeconomic disparities in white matter macrostructure and microstructure. We further investigated relations between family SES, children's white matter volume and integrity in tracts supporting EF, and performance on EF tasks.

**Results:**

Socioeconomic status was associated with fractional anisotropy (FA) and volume in multiple white matter tracts. Additionally, family income moderated the relation between white matter structure and cognitive flexibility. Specifically, across multiple tracts of interest, lower FA or lower volume was associated with reduced cognitive flexibility among children from lower income families. In contrast, children from higher income families showed preserved cognitive flexibility in the face of low white matter FA or volume. SES factors did not mediate or moderate links between white matter and either working memory or inhibitory control.

**Conclusions:**

This work adds to a growing body of literature suggesting that the socioeconomic contexts in which children develop not only shape cognitive functioning and its underlying neurobiology, but may also shape the relations between brain and behavior.

## 1 Introduction

Socioeconomic disparities in children's academic and cognitive outcomes have been well documented (McLoyd, 1998; Sirin, 2005) and current research has moved toward identifying potential neural underpinnings of these disparities. Differences in experiences, such as stress, language exposure, and cognitive stimulation, are differentially associated with growing up in more or less well‐resourced households; these differences in experience likely shape the development of brain areas that are important for children's cognitive skills (Brito & Noble, 2014; Noble, Houston, Kan, & Sowell, 2012). Consistent with this theoretical perspective, several studies have demonstrated associations between socioeconomic status (SES) and cortical and subcortical gray matter structure in children (see Brito & Noble, 2014 for a review). For example, differences have been reported in left hemisphere language regions including the left superior temporal gyrus, left inferior frontal gyrus, and left fusiform (Noble, Wolmetz, Ochs, Farah, & McCandliss, 2006; Noble, Houston, et al., 2012; Noble, et al., 2015; Raizada, Richards, Meltzoff, & Kuhl, 2008); the hippocampus, which supports memory, (Hanson, Chandra, Wolfe, & Pollak, 2011; Jednoróg et al., 2012; Luby et al., 2013; Noble, Houston, et al., 2012; Noble, Grieve, et al., 2012; Noble, et al., 2015); the prefrontal cortex which supports executive functioning, (Gianaros et al., 2007; Lawson, Duda, Avants, Wu, & Farah, 2013; Noble et al., 2015); and the amygdala which supports social‐emotional processing, (Gianaros et al., 2008; Luby et al., 2013; Noble, Houston, et al., 2012).

Few studies, however, have investigated the ways in which the context of low socioeconomic status may shape the macro‐ and microstructural properties of white matter in children. Jednoróg et al. (2012) found that SES, as measured by the Hollingshead index, was unrelated to white matter microstructure in a sample of 23 children. A larger sample of twins also did not find any direct relations between SES and white matter integrity as measured by fractional anisotropy (FA) (Chiang et al., 2011). Interestingly, however, the authors did find that SES modified the heritability of FA such that FA was more heritable among higher SES children. A related literature has shown that early deprivation in the form of institutionalization is associated with alterations in both white matter macrostructure, as measured by volume (Sheridan, Fox, Zeanah, McLaughlin, & Nelson, 2012) as well as white matter microstructure, or integrity (Bick et al., 2015). Moreover, one study has demonstrated relations between one's own educational achievement in young adulthood and integrity in the superior longitudinal fasciculus and cingulum bundle, such that higher education was associated with lower FA in those two regions (Noble, Korgaonkar, Grieve, & Brickman, 2013).

Much of the work on SES differences in children's brain structure and function has focused on the development of executive functions, in part because the protracted development of brain areas that support these processes likely makes them more susceptible to environmental influence (Giedd, 2004; Gogtay et al., 2004; Huttenlocher, 1979; Klingberg, Vaidya, Gabrieli, Moseley, & Hedehus, 1999; Liston et al., 2006; Sowell et al., 2003, 2004). Executive functions (EF) are cognitive skills involved in planning and goal‐directed behavior and play an important role in academic achievement and school success (Blair, 2002). Several studies have demonstrated that SES is related to the development of most aspects of executive functioning including working memory, inhibitory control, and cognitive flexibility (Blair et al., 2011; Farah et al., 2006; Noble, McCandliss, & Farah, 2007; Sarsour et al., 2011). Moreover, a growing body of work has found associations between SES and both function and structure of brain areas that underlie executive function capabilities. In one functional magnetic resonance imaging (fMRI) study that used a complex stimulus‐response learning task that elicits prefrontal activation in adults, lower SES children performed more poorly than their higher SES counterparts. They were also more likely to activate the right medial frontal gyrus as compared to higher SES children, possibly reflecting less efficient processing (Sheridan, Sarsour, Jutte, D'Esposito, & Boyce, 2012). In adults, a study of functional connectivity of corticostriatal brain systems during a reward processing task found that lower parental education was associated with reduced functional connectivity of perigenual anterior cingulate cortex (pACC) and orbitofrontal cortex (OFC) regions to the dorsomedial prefrontal cortex (dMPFC) and ventral striatum, even after controlling for participants’ own (adult) level of education (Gianaros et al., 2011). Additional studies have reported SES‐related differences in structural properties of gray matter areas that support executive function. In a study of gray matter development from infancy through early childhood, lower family income was associated with smaller frontal lobe volumes in infancy and with slower growth of the frontal lobes through early childhood (Hanson et al., 2013). Higher parental education has also been associated with greater cortical thickness in specific frontal regions including the right anterior cingulate gyrus and left superior frontal gyrus in children (Lawson et al., 2013). In adults, subjective social status has been associated with gray matter volume in the anterior cingulate cortex, an area important for cognitive control (Gianaros et al., 2007). In a prior study with the same cohort as analyzed in the present paper, family income was logarithmically related to differences in cortical surface area, and surface area partially accounted for links between family income and certain executive function skills (Noble et al., 2015).

Diffusion tensor imaging (DTI) studies have identified several white matter tracts that appear to be important for performance on executive functioning tasks. These include the cingulum bundle (CB) (Kantarci et al., 2011; Konrad et al., 2010; Liston et al., 2006; Makris et al., 2008; Murphy et al., 2007; Pavuluri et al., 2009; Peters et al., 2014; Schermuly et al., 2010; Skranes et al., 2009); the superior longitudinal fasciculus (SLF) (Ashtari et al., 2007; Burzynska et al., 2011; Charlton, Barrick, Lawes, Markus, & Morris, 2010; Karlsgodt et al., 2008; Kennedy & Raz, 2009; Konrad et al., 2010; Liston, Cohen, Teslovich, Levenson, & Casey, 2011; Makris et al., 2008; Olesen, Nagy, Westerberg, & Klingberg, 2003; Pavuluri et al., 2009; Perry et al., 2009; Sasson, Doniger, Pasternak, Tarrasch, & Assaf, 2013; Vestergaard et al., 2011); the anterior thalamic radiations (ATR) (Liston et al., 2011; Niogi et al., 2008; Pavuluri et al., 2009); and the inferior longitudinal fasciculus (ILF) (Perry et al., 2009; Sarro et al., 2011; Takeuchi et al., 2013). The CB, SLF, and ATR all have projections to the anterior cingulate gyrus (Makris et al., 2008; Niogi et al., 2008; Schermuly et al., 2010) a prefrontal region long recognized to support executive functioning (Adleman et al., 2002; Botvinick, Braver, Barch, Carter, & Cohen, 2001; Bush et al., 1998; Casey et al., 2000), and the ILF connects occipital and temporal regions which support visual memory processes (Catani, Jones, Donato, & Ffytche, 2003; Perry et al., 2009). Studies of white matter tracts have investigated both macrostructural properties, such as volume, as well as microstructural properties including integrity as measured by FA. FA is an important microstructural property that is thought to indicate greater efficiency in information transfer across the brain, and recent work demonstrates that declines in FA in late adulthood have been linked to declines in fluid intelligence (Ritchie et al., 2015).

Few studies, however, have investigated the role that macro‐ and microstructural properties of white matter tracts may play in the relation between SES and children's EF. In considering relations across SES, brain structure, and executive function, both mediation and moderation pathways have been proposed (Brito & Noble, 2014; Noble, Houston, et al., 2012; Ursache & Noble, 2016). In a mediating model, differences in white matter structure are hypothesized to account for the links between SES and behavioral performance on executive function tasks. Some support for this pathway comes from a study by Noble et al. (2013) which found that the relation between young adults’ education levels and performance on a Stroop‐like cognitive control task was mediated by integrity of the SLF and CB white matter tracts. Similarly, Noble et al. (2015) found that differences in cortical surface area partially mediated relations between family income and performance on the flanker inhibitory control task and on the working memory task.

In a moderating model, SES is hypothesized to interact with brain structure such that the relation of brain structure to cognitive function would differ across SES. This model stems from several areas of research that suggest that brain‐behavior relations may differ for children from different SES backgrounds (for a review see Ursache & Noble, 2016). For example, some evidence suggests that children from higher SES families who are at‐risk for reading difficulties may be able to develop good reading skills despite atypical activation in systems that are classically important for reading development (Noble, Wolmetz, et al., 2006; Shaywitz et al., 2003). Such studies suggest that experiences associated with higher SES backgrounds may buffer against risk for poorer cognitive performance. Moreover, this model is related to the theory of cognitive reserve which states that, because of differences in lifetime experience, higher SES individuals may be better able to call upon other neurocognitive resources and/or alter neurocognitive processing such that brain pathology does not result in otherwise expected cognitive deficits (Stern, 2009). For example, higher SES older adults may be able to recruit additional neural resources to buffer against some of the typical age‐related memory decline (Czernochowski, Fabiani, & Friedman, 2008). Thus, it is possible that the relation between white matter micro‐ or macrostructure and performance on executive functioning tasks may differ for children from lower versus higher SES backgrounds.

### 1.1 Current study

This study examines the relations between SES, white matter structure, and executive functioning. We first examine socioeconomic disparities in two facets of white matter structure— macrostructure as measured by volume, and microstructure as measured by fractional anisotropy (FA), an indicator of tract integrity—across the brain. Next, we investigate whether differences in white matter micro‐ or macrostructure in four a priori tracts of interest, namely the CB, ILF, SLF, and ATR, mediate or moderate SES disparities in executive function.

## 2 Methods

### 2.1 Participants

Data used in the preparation of this article were obtained from the Pediatric Imaging, Neurocognition and Genetics (PING) Study database (RRID:SCR_008953; http://ping.chd.ucsd.edu/). PING was launched in 2009 by the National Institute on Drug Abuse (NIDA) and the Eunice Kennedy Shriver National Institute of Child Health and Human Development (NICHD) as a 2‐year project of the American Recovery and Reinvestment Act. The primary goal of PING has been to create a data resource of highly standardized and carefully curated magnetic resonance imaging (MRI) data, comprehensive genotyping data, and developmental and neuropsychological assessments for a large cohort of developing children aged 3–20 years. The scientific aim of the project is, by openly sharing these data, to amplify the power and productivity of investigations of healthy and disordered development in children, and to increase understanding of the origins of variation in neurobehavioral phenotypes. For up‐to‐date information, see http://ping.chd.ucsd.edu/.

Participants were recruited through a combination of web‐based, word‐of‐mouth, and community advertising at nine university‐based data collection sites in and around the cities of Los Angeles, San Diego, New Haven, Sacramento, San Diego, Boston, Baltimore, Honolulu, and New York. Participants were excluded if they had a history of neurological, psychiatric, medical, or developmental disorders. All participants and their parents gave their informed written consent/assent to participate in all study procedures, including whole genome SNP genotype, neuropsychological assessments (NIH Toolbox Cognition Battery; RRID:SCR_002423; Akshoomoff et al., 2014), demographic and developmental history questionnaires, and high‐resolution brain MRI. Each data collection site's Office of Protection of Research Subjects and Institutional Review Board approved the study. The sample for the current study was limited to the 1082 participants with complete data on imaging measures, age, sex, and genetic ancestry factors. Sample demographics are shown in Table 1.

**Table 1:** Descriptive statistics

	*N*	Mean or %	SD	Range
Age	1082	12.21	4.91	3–21
Sex (male = 1)	1082	52%		
Family Income	1036	97209	76233	4500–325000
Parental Education	1047	15.00	2.26	6–18
GAF Africa	1082	0.13	0.27	0–1
GAF American Indian	1082	0.05	0.12	0–0.83
GAF East Asian	1082	0.16	0.31	0–1
GAF Oceanic	1082	0.01	0.03	0–0.25
GAF Central Asian	1082	0.03	0.14	0–1
GAF European	1082	0.63	0.374	0–1
List Sort Working Memory	1069	17.98	5.25	0–28
DCCS Cognitive Flexibility	979	7.75	1.45	2–10
Flanker Inhibitory Control	1059	7.74	1.79	1–10

### 2.2 Measures

#### 2.2.1 Socioeconomic status

Parents were asked to report the level of educational attainment for each parent in the home. The average parental educational attainment was used in all analyses. Parents were also asked to report the total yearly family income. Data were not collected on the number of adults and children in the home, and thus we could not calculate income‐to‐needs ratios. Both family income and parental education data were originally collected in bins, which were recorded as the means of the bins for analysis. Family income was log transformed for all analyses due to the typically observed positive skew. As expected, family income and parental education were highly correlated (*r *=* *.546, *p *<* *.001).

#### 2.2.2 Image acquisition and Processing

For complete details of the image acquisition and processing methods used in the creation of this publicly available dataset, please see Fjell et al. (2012) and Jernigan et al. (2016). Briefly, across the nine sites and 12 scanners, a standardized multiple‐modality high‐resolution structural MRI protocol was implemented, including a conventional three‐plane localizer, a sagittal three‐dimensional inversion recovery spoiled gradient echo T1‐weighted volume optimized for maximum gray/white matter contrast (echo time = 3.5 ms, repetition time = 8.1 ms, inversion time = 640 ms, flip angle = 8°, receiver bandwidth = ±31.25 kHz, FOV = 24 cm, frequency = 256, phase = 192, slice thickness = 1.2 mm), and a two axial two‐dimensional diffusion tensor imaging (DTI) pepolar scans (30‐directions b‐value = 1,000, TE = 83 ms, TR = 13,600 ms, frequency = 96, phase = 96, slice thickness = 2.5 mm). Scanning duration for DTI was 4:24. The scanner models used at each site can be found in Fjell et al., 2012. Pooling of data from different scanners imposes challenges, although the sequences were optimized for yielding comparable results, and scanner is included as the covariate in all statistical analyses.

Diffusion‐weighted images were corrected for eddy current distortion using a least squares inverse and iterative conjugate gradient descent method to solve for the 12 scaling and translation parameters describing eddy current distortions across the entire diffusion MRI scan, explicitly taking into account the orientations and amplitudes of the diffusion gradient (Zhuang et al., 2006). Head motion was corrected by registering each diffusion‐weighted image to a corresponding image synthesized from a tensor fit to the data (Hagler et al., 2009). Diffusion MRI data were corrected for spatial and intensity distortions caused by B0 magnetic field in homogeneities using the reversing gradient method (Holland, Kuperman, & Dale, 2010). Distortions caused by gradient nonlinearities were corrected by applying a predefined, scanner‐specific, nonlinear transformation (Jovicich et al., 2006). Diffusion‐weighted images were automatically registered to T1‐weighted structural images using mutual information (Wells, Viola, Atsumi, Nakajima, & Kikinis, 1996) and rigidly resampled into a standard orientation relative to the T1‐weighted images with isotropic 2‐mm voxels. Cubic interpolation was used for all resampling steps.

Diffusion parameters were computed for major brain fiber tracts. AtlasTrack was used to automatically label long‐range white matter tracts based on a probabilistic atlas of fiber tract locations and orientations (Hagler et al., 2009). The fiber atlas contains prior probabilities and orientation information for specific long‐range projection fibers, including some additional fiber tracts not included in the original description, such as corticostriate connections and inferior to superior frontal corticocortical connections (Jernigan et al., 2016). Fiber tract volumes of DTI Atlas tracts were computed and conventional DTI methods were used to calculate fractional anisotropy (FA; (Basser, Mattiello, & LeBihan, 1994; Pierpaoli, Jezzard, Basser, Barnett, & Di Chiro, 1996) which represents the degree of directionality of random water diffusion.

#### 2.2.3 Flanker inhibitory control test

The NIH Toolbox Cognition Battery version of the flanker task was adapted from the Attention Network Test (ANT; Rueda et al., 2004). Participants were presented with a stimulus on the center of a computer screen and were required to indicate the left–right orientation while inhibiting attention to the flankers (surrounding stimuli). On some trials, the orientation of the flankers was congruent with the orientation of the central stimulus, and on the other trials, the flankers were incongruent. The test consisted of a block of 25 fish trials (designed to be more engaging and easier to see to make the task easier for children) and a block of 25 arrow trials, with 16 congruent and 9 incongruent trials in each block, presented in pseudorandom order. Participants who responded correctly on five or more of the nine incongruent trials then proceeded to the arrows block. All children aged 9 and above received both the fish and arrows blocks regardless of performance. The inhibitory control score was based on performance on both congruent and incongruent trials. A two‐vector method was used that incorporated both accuracy and reaction time (RT) for participants who maintained a high level of accuracy (>80% correct), and accuracy only for those who did not meet this criterion. Each vector score ranged from 0 to 5, for a maximum total score of 10. The accuracy vector score was calculated by multiplying the number of correct responses by 0.125. The reaction time vector score was generated using individuals’ raw median reaction time score for the incongruent condition. Median reaction time values were computed using only correct trials with reaction times greater than or equal to 100 ms and reaction times no larger than 3 SDs away from the individual's mean (for respective trial type). Lower reaction times result in higher vector scores. For further details on how reaction times were converted to the vector score, please see National Institutes of Health Toolbox Cognition Battery (NIH Toolbox CB) (2013).

#### 2.2.4 List sorting working memory test

This working memory measure requires participants to order stimuli by size (Tulsky et al., 2013). Participants were presented with a series of pictures on a computer screen and heard the name of the object from a speaker. The test was divided into the one‐list and two‐list conditions. In the one‐list condition, participants were told to remember a series of objects (food or animals) and repeat them in order, from smallest to largest. In the two‐list condition, participants were told to remember a series of objects (food and animals, intermixed) and then again report the food in order of size, followed by animals in order of size. Working memory scores consisted of combined total items correct on both one‐list and two‐list conditions, with a maximum of 28 points.

#### 2.2.5 Dimensional change card sort task

The dimensional change card sort task (DCCS) is a test of cognitive flexibility taken from the NIH Toolbox. Participants are shown two target pictures, one on each side of the screen, that vary along two dimensions (e.g., shape and color). Participants are asked to match a series of bivalent test pictures (e.g., yellow balls and blue trucks) to the target pictures, first according to one dimension (e.g., color) and then, after a number of trials, according to the other dimension (e.g., shape). “Switch” trials are also employed, in which the participant must change the dimension being matched. For example, after four straight trials matching on shape, the participant may be asked to match on color on the next trial and then go back to shape, thus requiring the cognitive flexibility to quickly choose the correct stimulus. A two‐vector scoring method was used that incorporated both accuracy and reaction time (RT) for participants who maintained a high level of accuracy (>80% correct), and accuracy only for those who did not meet this criteria. Each vector score ranged from 0 to 5, for a maximum total score of 10. The accuracy vector score was calculated by multiplying the number of correct responses by 0.125. The reaction time vector score was generated using individuals’ raw, median reaction time score during the nondominant dimension condition. Median reaction time values were computed using only correct trials with reaction times greater than or equal to 100 ms and reaction times no larger than 3 SDs away from the individual's mean (for respective trial type). Lower reaction times result in higher vector scores. For further details on how reaction times were converted to the vector score, please see National Institutes of Heatlh Toolbox Cognition Battery (NIH Toolbox CB) (2013).

#### 2.2.6 Genetic collection and analysis

Saliva samples were sent to Scripps Translational Research Institute (STRI) for analysis. Once extracted, genomic DNA was genotyped with Illumina Human660W‐Quad BeadChip. Replication and quality control filters (that is, sample call rate >99, call rates >95%, minor allele frequency >5%) were performed (Bakken et al., 2012). To assess genetic ancestry and admixture proportions in the PING participants, a supervised clustering approach implemented in the ADMIXTURE software was used (Alexander & Lange, 2011). Using this approach, a genetic ancestry factor (GAF) was developed for each participant, representing the proportion of ancestral descent for each of six major continental populations: African, Central Asian, East Asian, European, Native American, and Oceanic. Implementation of ancestry and admixture proportions in the PING subjects is described elsewhere (Fjell et al., 2012). A more complete description of the genetic ancestry of the PING sample is also presented elsewhere (Akshoomoff et al., 2014).

### 2.3 Analysis plan

**Figure 1:**
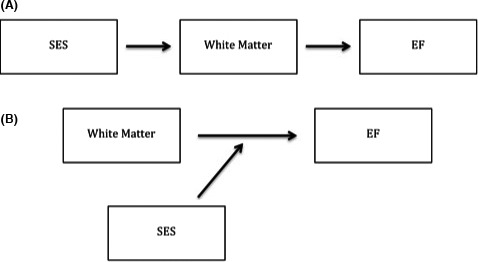
Hypotheses. (A) The relation of SES to executive function may be mediated by white matter structure. (B) Alternatively, SES may moderate the relation of white matter structure to executive function

To investigate SES differences in white matter micro‐ and macrostructure, we first examined the extent to which family income and education are related to white matter FA and volume across the brain. For these analyses, we used a Bonferroni‐adjusted alpha of 0.0013 (i.e., 0.05/39 tracts). Next, to investigate the role that white matter structure plays in the relation of SES to EF, we tested mediating and moderating hypotheses regarding four tracts, chosen a priori*,* that have been shown to be important for EF (the CB, SLF, ILF, ATR). As we did not have a priori hypotheses regarding laterality, we averaged FA and summed volume across left and right hemispheres for these analyses. To test for mediation, we examined whether differences in volume or integrity of those tracts attenuate (i.e., statistically mediate) associations between family income/parental education and EF. Mediation analyses were conducted using the INDIRECT Macro (Preacher & Hayes, 2008) with 5000 bootstrap samples to test the significance of indirect effects. To test for moderation, we examined whether SES factors (parent education/family income) altered (i.e., statistically moderated) associations between white matter tract volume/integrity and EF (see Fig. 1). Interaction terms were created by multiplying parent education or family income by volume or integrity of the white matter tracts of interest. Linear regression was then used to test the significance of the interaction term in predicting EF. For each hypothesis, our Bonferroni‐adjusted alpha was set at 0.0125, to adjust for comparisons in four tracts. We additionally conducted exploratory, unadjusted analyses of the same relations across all fibers.

Analyses predicting white matter integrity included as covariates age, age squared, sex, genetic ancestry, and scanner. Analyses predicting white matter volume included as covariates age, whole brain volume, sex, genetic ancestry, and scanner. Analyses predicting EF included as covariates age, age squared, sex, and genetic ancestry.

The sample was limited to the 1082 participants with complete data on imaging measures, age, sex, and genetic ancestry factors. Analyses were conducted for all participants with complete data on the variables included in a given analysis. Descriptive statistics and sample sizes for each variable are shown in Table 1. Analyses were conducted in SPSS (version 22).

## 3 Results

### 3.1 Relations of SES to white matter integrity and volume

Higher family income was related to higher FA in the right parahippocampal cingulum (β = 0.101, *p = *.001) and the right superior corticostriate tract in the frontal cortex (β = 0.095, *p *=* *.001). There were no significant relations between family income and white matter volume. Higher parental education was related to higher FA in the left superior cortiostriate tract in the parietal cortex (β = 0.088, *p *=* *.001). Higher parental education was associated with lower white matter volume in the left inferior frontal superior frontal cortex tract (β = −0.063, *p *<* *.001). Tables S1–S4 show uncorrected relations between SES and white matter FA and volume in all tracts across the brain.

### 3.2 SES associations with EF mediated by white matter integrity

To begin examining whether white matter structure mediated pathways between SES and EF, we first replicated results demonstrating links between SES and children's EF. As shown in Table 2, when adjusting for age, age squared, sex, and genetic ancestry, higher family income was significantly related to higher scores on the cognitive flexibility (β = 0.049, *p *=* *.021), working memory (β = 0.058, *p *=* *.003), and inhibitory control (β = 0.039, *p *=* *.045) tasks. As shown in Table 2, when adjusting for age, age squared, sex, and genetic ancestry, higher parental education was significantly related to higher scores on the cognitive flexibility, (β = 0.072, *p *=* *.001), working memory, (β = 0.095, *p *<* *.001), and inhibitory control (β = 0.073, *p *<* *.001) tasks.

**Table 2:** Relation of family income and parental education to executive function

Model 1	DCCS (*n *= 935)			List Sort (*n *= 1023)			Flanker (*n *= 1013)		
	β	*t*	*p*‐value	β	*t*	*p*‐value	β	*t*	*p*‐value
Age	2.757	25.326	<.001	2.842	29.480	<.001	3.011	30.898	<.001
Age sq	−2.077	−19.063	<.001	−2.171	−22.526	<.001	−2.346	−24.051	<.001
Sex	−0.053	−2.781	.006	0.043	2.441	.015	0.014	0.802	.423
GAF Africa	−0.046	−2.141	.033	−0.101	−5.202	<.001	−0.037	−1.868	.062
GAF American Indian	−0.059	−2.936	.003	‐0.026	−1.416	.157	−0.031	−1.685	.092
GAF East Asian	0.023	1.065	.287	−0.061	−3.011	.003	0.030	1.472	.141
GAF Oceanic	−0.052	−2.440	.015	−0.017	−0.869	.385	−0.035	−1.761	.078
GAF Central Asian	0.007	0.342	.732	0.008	0.449	.654	0.014	0.747	.455
Income	0.049	2.310	.021	0.058	2.957	.003	0.039	2.006	.045
	Adjusted *R* ^2* *^= .662			Adjusted *R* ^2^ * *= .687			Adjusted *R* ^*2*^ * *= .687		

Model 2	DCCS (*n *= 945)			List Sort (*n *= 1034)			Flanker (*n *= 1024)		
	β	*t*	*p*‐value	β	*t*	*p*‐value	β	*t*	*p*‐value
Age	2.811	26.212	<.001	2.876	30.621	<.001	3.048	32.119	<.001
Age sq	−2.128	−19.875	<.001	−2.196	−23.439	<.001	−2.376	−25.074	<.001
Sex	−0.052	−2.729	.006	0.044	2.511	.012	0.012	0.692	.489
GAF Africa	−0.040	−1.970	.049	−0.095	−5.090	<.001	−0.028	−1.474	.141
GAF American Indian	−0.043	−2.085	.037	−0.011	−0.596	.551	−0.014	−0.748	.455
GAF East Asian	0.013	0.611	.541	−0.059	−2.970	.003	0.030	1.514	.13
GAF Oceanic	−0.047	−2.216	.027	−0.010	−0.493	.622	−0.025	−1.300	.194
GAF Central Asian	0.013	0.665	.506	0.007	0.420	.675	0.011	0.613	.54
Education	0.072	3.441	.001	0.095	4.956	<.001	0.073	3.798	<.001
	Adjusted *R* ^2^ * *= .665			Adjusted *R* ^2^ * *= .695			Adjusted *R* ^2^ * *= .695		

The CB, ILF, SLF, and ATR were hypothesized a priori to play a role in mediating the links between SES and EF. We therefore next examined whether parental education and/or family income was related to FA in these tracts. Family income was inversely related to ATR FA (β = −0.063, *p *=* *.014) and parental education was positively related to ILF FA (β = 0.056, *p *=* *.013). Although these relations did not reach statistical significance at the Bonferroni‐adjusted levels, they were borderline significant. We therefore next examined whether FA in either of these tracts was associated with performance on each of the three EF tasks. Higher FA in the ILF was associated with higher inhibitory control (β = 0.067, *p *=* *.011). FA in the ATR was not associated with performance on any of the EF tasks.

Finally, we tested whether FA in the ILF mediated the association between parental education and inhibitory control. Results indicated that the indirect effect of parental education on inhibitory control through ILF FA was not significant (98.75% CI: [−.0007,.0094]).

### 3.3 Moderation of SES effects on EF by white matter volume and integrity

We next examined whether SES‐moderated relations between white matter structure in the four a priori tracts of interest and performance on each of the EF tasks.

**Figure 2:**
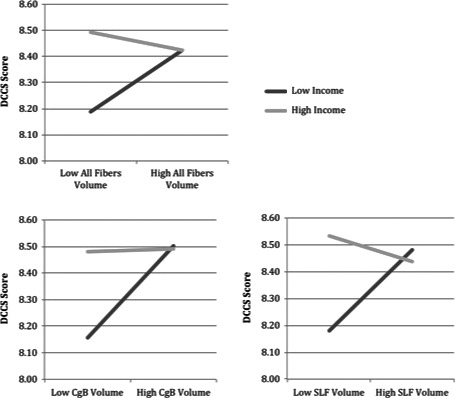
Family income moderates the association between white matter volume and performance on the DCCS cognitive flexibility task. Model‐estimated simple slopes of relations between white matter volume and DCCS performance are plotted at values of 1 standard deviation above and below the mean family income level

As shown in Table 3, for the DCCS task, there was a significant volume x family income interaction in two of the a priori tracts as well as across the whole brain: CB (β = −0.718, *p *=* *.002), SLF (β = −0.915, *p *<* *.001), and all fibers (β = −0.741, *p *=* *.007). There were borderline significant interactions in each of the other a priori tracts: ILF (β = −0.629, *p *=* *.016), ATR (β = −0.602, *p *=* *.022). Thus family income moderated the association between white matter volume and cognitive flexibility. As shown in Fig. 2, the pattern of moderation largely suggests that for children from lower income families, lower white matter volume is associated with reduced cognitive flexibility, whereas children from higher income families tended to show higher levels of cognitive flexibility regardless of white matter volume.

**Table 3:** Income moderates the relation between volume and cognitive flexibility (*n *= 935)

	β	*t*	*p*‐value
CB volume			
Adjusted *R* ^2^ * *= .668			
CB volume	0.676	3.314	.001
Income	0.370	3.406	.001
CB * Income	−0.718	−3.033	.002
SLF volume			
Adjusted *R* ^2^ * *= .668			
SLF volume	0.780	3.641	<.001
Income	0.499	3.880	<.001
SLF * Income	−0.915	−3.564	<.001
ILF volume			
Adjusted *R* ^2^ * *= .667			
ILF volume	0.421	1.963	.050
Income	0.360	2.747	.006
ILF *Income	−0.629	−2.410	.016
ATR volume			
Adjusted *R* ^2^ * *= .665			
ATR volume	0.486	2.299	.022
Income	0.379	2.592	.010
ATR * Income	−0.602	−2.292	.022
All fibers volume			
Adjusted *R* ^2^ * *= .666			
All Fibers Volume	0.593	2.753	.006
Income	0.470	2.998	.003
All Fibers * Income	−0.741	−2.720	.007

**Figure 3:**
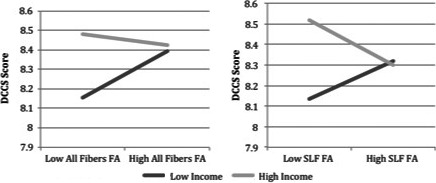
Family income moderates the association between white matter FA and performance on the DCCS cognitive flexibility task. Model‐estimated simple slopes of relations between white matter FA and DCCS performance are plotted at values of 1 standard deviation above and below the mean family income level

As shown in Table 4, for the DCCS task, there was a significant FA x family income interaction in the SLF (β = −1.315, *p *=* *.001) as well as across the whole brain (β = −1.086, *p *=* *.012), and there were borderline significant interactions in two other a priori tracts: CB (β = −0.729, *p *=* *.013), ILF (β = −0.855, *p *=* *.017). Thus, family income moderates the associations between FA and cognitive flexibility. As shown in Fig. 3, the pattern of moderation largely suggests that for children from lower income families, lower white matter integrity is associated with lower cognitive flexibility, whereas children from higher income families tend to exhibit higher cognitive flexibility regardless of their white matter microstructure.

**Table 4:** Income moderates the relation of FA to cognitive flexibility (*n *= 935)

	β	*t*	*p*‐value
CB FA			
Adjusted *R* ^2^ * *= .665			
CB FA	0.579	2.577	.010
Income	0.529	2.729	.006
CB * Income	−0.729	−2.486	.013
SLF FA			
Adjusted *R* ^2^ * *= .667			
SLF FA	0.741	3.384	.001
Income	1.112	3.575	<.001
SLF * Income	−1.315	−3.418	.001
ILF FA			
Adjusted *R* ^2^ * *= .665			
ILF FA	0.536	2.323	.020
Income	0.686	2.567	.010
ILF * Income	−0.855	−2.383	.017
All Fibers FA			
Adjusted *R* ^2^ * *= .666			
All Fibers FA	0.584	2.630	.009
Income	0.998	2.646	.008
All Fibers * Income	−1.086	−2.516	.012

Because of the negatively skewed distribution of the DCCS, we reran the analyses after winsorizing scores on the task by recoding any values that were more than 3 SD below the mean to the value of 3 SD below the mean. The pattern of results remained the same.

No other relations between white matter macro‐ or microstructure and EF task performance were moderated by SES factors at Bonferroni‐adjusted levels of significance.

## 4 Discussion

This study is the largest investigation to date of SES differences in macro‐ and microstructure of white matter tracts. Additionally, it is the first to report associations between parental SES and children's white matter integrity and volume. Specifically, we found that higher family income was related to higher FA in two regions: the right parahippocampal cingulum, which is a limbic tract involved in memory (Zhuang et al., 2013) and which is part of the cingulum bundle important for EF; and the right superior corticostriate tract in the frontal cortex, which connects the cortex to the striatum involved in reward processing. Additionally, higher parental education was related to higher integrity (FA) in the left superior corticostriate in the parietal cortex. Higher parental education was associated with lower white matter volume in the left inferior frontal superior frontal cortex tract, which has been associated with language function (Kucukboyaci et al., 2014).

These results add to an emerging body of work, which has demonstrated SES‐related differences in children's brain structure and function (see Brito & Noble, 2014 and Ursache & Noble, 2016). Little is known, however, about the specific mechanistic pathways through which SES might affect white matter development. Differences in experiences of family stress and cognitive stimulation are candidates for investigation, as these have been linked to socioeconomic differences in gray matter structure and function (Brito & Noble, 2014; Noble, Houston, et al., 2012; Noble, Grieve, et al., 2012). Socioeconomic disadvantage may lead to increased experience of stress through multiple pathways including both physical and social characteristics of the environment (Evans, 2004). Lower SES homes are often characterized by harsher parenting, crowding, noise, chaotic schedules, a lack of routines, and a generally higher level of unpredictability, all of which can contribute to an increase in stress in children (Adler & Snibbe, 2003; Combs‐Orme & Cain, 2006; Evans, Gonnella, Marcynyszyn, Gentile, & Salpekar, 2005). Children from lower SES families are also more likely to be exposed to environments that are less cognitively stimulating and linguistically enriched (see Perkins et al., 2013 for a review). For example, children from lower SES families are exposed to fewer words (Hart & Risley, 1995) and less complex sentences (Hoff, 2003; Huttenlocher, Vasilyeva, Cymerman, & Levine, 2002), and differences in child‐directed speech have been associated with children's language abilities (Weisleder & Fernald, 2013). How exactly these or other experiences might translate into white matter differences, however, needs to be explored in future work.

This is also the first study to explore the ways in which children's socioeconomic context and white matter structure might explain behavioral performance on executive function tasks. We first investigated the extent to which differences in white matter structure in four a priori tracts of interest might account for, or mediate, the links between socioeconomic factors and EF performance. In doing so, we first replicated prior behavioral findings that higher family income and parental education were associated with better performance on working memory, inhibitory control, and cognitive flexibility tasks. These results are in line with past research demonstrating that executive functions are impaired among children growing up in lower SES contexts (Blair et al., 2011; Farah et al., 2006; Noble et al., 2007; Sarsour et al., 2011). In testing mediation pathways, however, we did not find evidence for our hypothesis that white matter structure may mediate the relation between SES and EF. As such, we did not replicate results from a prior study which had found mediation pathways in which FA in the SLF and CB accounted for links between young adults’ education levels and their performance on a Stroop task of cognitive control (Noble et al., 2013). The difference in participants’ ages between the two studies may have played a role, or it may be that participants’ own levels of education have a broader relation to white matter microstructure because they are a more direct indicator of participants’ own experiences.

Finally, we examined the hypothesis that relations between brain and behavior may differ as a function of family socioeconomic background. We found that family income moderated the relations between white matter microstructure (FA in the SLF, and across the whole brain) and cognitive flexibility. A similar pattern of borderline significant results was found in the CB and ILF. Additionally, family income moderated the relations between white matter macrostructure (volume in the CB, SLF, and across the whole brain) and cognitive flexibility. A similar pattern of borderline significant results was found in the ILF and ATR. The pattern of results largely suggested that lower integrity or volume of white matter was associated with lower cognitive flexibility for children from lower income families, but that children with similarly low white matter micro‐ or macrostructure from higher SES families were buffered against these effects on cognitive flexibility. These findings are consistent with a body of research indicating that higher SES may serve to buffer children and adults from a range of potentially detrimental cognitive outcomes (Bellinger, Leviton, Waternaux, Needleman, & Rabinowitz, 1988; Czernochowski et al., 2008; Harvey, Hamlin, Kumar, & Delves, 1984; Noble, Farah, & McCandliss, 2006; Rauh et al., 2004; Shaywitz et al., 2003; Tong, McMichael, & Baghurst, 2000; Winneke & Kraemer, 1984). For example, Noble, Farah et al. (2006) found that among lower SES struggling readers, phonological skill differences were associated with large differences in brain activation during a reading task, but that this brain‐behavior relationship weakened as SES increased. That is, children who struggled with reading in the context of limited access to resources showed *typical* brain‐behavior relationships, whereas children who struggled with reading despite plentiful access to resources showed *atypical* brain‐behavior relationships. One possible interpretation of our moderation finding is that higher income families may be able to devote more resources to fostering their children's cognitive skills, and/or are better able to devote extra resources to helping children who exhibit cognitive difficulties or impairments. It is possible that such increased access to resources may enable children to use different neural resources or strategies in order to exhibit a high level of behavioral performance.

Interestingly, however, the moderation findings were only significant for one aspect of executive function, namely cognitive flexibility, and were not significant for working memory or inhibitory control. Cognitive flexibility differs from the other two aspects of executive function in that it is a more complex and global process that develops later and builds on working memory and inhibitory control skills (Diamond, 2013). Although we did not hypothesize that our results would extend only to cognitive flexibility, it may be that its more global nature as an aspect of executive function plays a role in these findings.

Moreover, all of the significant SES × white matter structure interactions reported here were found for family income but not for parental education. The extent to which these different components of SES may be differentially associated with specific neurocognitive outcomes is only beginning to be explored. Although we did not have a priori hypotheses about the effects of family income versus parental education, it may be that higher family income plays a particularly salient role in giving families the resources to purchase better housing, child care, learning opportunities, tutoring services, extracurricular activities, and medical services that could promote executive function development among those who might otherwise be at risk for impairments.

## 5 Limitations and Future Directions

The tract‐based approach that we utilized in the present study is limited in that tract size may play a role in our ability to detect relations between SES and white matter. For example, in larger tracts, significant relations in one part of the tract may be washed out by nonsignificant relations elsewhere in the tract. Future work should consider voxel‐based approaches to overcome this limitation. Our results exploring the role that white matter structure plays in the relation of SES to EF are additionally limited by the small effect sizes, and future work is needed to replicate these findings. Moreover, it is unclear why family income moderated the effects of white matter integrity and volume primarily in the context of cognitive flexibility, and not in the context of other executive functions. This should be explored in future work.

## 6 Conclusions

This study is the largest examination of SES differences in white matter structure and is the first to investigate relations among family income and parental education, white matter integrity and volume, and executive functioning. Parental SES was related to white matter integrity and volume in multiple tracts. Additionally, SES was found to moderate the relation between white matter structure and cognitive flexibility. These results suggest that children from higher income families may be buffered from behavioral deficits that are typically associated with lower white matter volume and integrity. As such, this work adds to a growing body of literature suggesting that the socioeconomic contexts in which children develop not only shape cognitive functioning and its underlying neurobiology, but may also shape the relations between brain and behavior.

## Funding information

Teachers College, National Institutes of Health, (Grant/Award Number: ‘RC2DA029475’,’T32‐NS07153’) Annie E. Casey Foundation, W.K. Kellogg Foundation.

## Conflict of Interest

None declared.
