# Supplementary material for: Socioeconomic status, white matter, and executive function in children
Source: Brain Behav. 2016 Aug 2;6(10):e00531. doi: 10.1002/brb3.531 (PMC5064342; doi:10.1002/brb3.531)
Supplement: Supplementary file 1 [file BRB3-6-e00531-s001.docx]

**Supplementary Tables**

| Table I. Regression results of relations of family income to FA in each fiber tract (n = 1036). | | | | | |
| --- | --- | --- | --- | --- | --- |
|  | Beta | t | p-value |  | Adj R sq |
| R Fornix | 0.001 | 0.033 | .973 |  | .147 |
| L Fornix | -0.013 | -0.396 | .692 |  | .126 |
| R Cingulate Cingulum | -0.027 | -1.089 | .276 |  | .506 |
| L Cingulate Cingulum | -0.028 | -1.150 | .250 |  | .504 |
| R Parahippocampal Cingulum | 0.101 | 3.442 | .001 | * | .301 |
| L Parahippocampal Cingulum | 0.084 | 2.800 | .005 |  | .262 |
| R Corticospinal/Pyramidal | 0.066 | 2.274 | .023 |  | .313 |
| L Corticospinal/Pyramidal | 0.057 | 1.908 | .057 |  | .267 |
| R Anterior Thalamic Radiations | -0.050 | -1.939 | .053 |  | .468 |
| L Anterior Thalamic Radiations | -0.070 | -2.618 | .009 |  | .418 |
| R Uncinate | 0.008 | 0.325 | .745 |  | .454 |
| L Uncinate | -0.006 | -0.207 | .836 |  | .294 |
| R Inferior Longitudinal Fasciculus | 0.016 | 0.639 | .523 |  | .520 |
| L Inferior Longitudinal Fasciculus | 0.036 | 1.544 | .123 |  | .547 |
| R Inferior-Fronto-Occipital Fasciculus | 0.031 | 1.374 | .170 |  | .577 |
| L Inferior-Fronto-Occipital Fasciculus | 0.035 | 1.530 | .126 |  | .574 |
| Foreceps Major | 0.021 | 0.784 | .433 |  | .410 |
| Foreceps Minor | -0.048 | -1.560 | .119 |  | .248 |
| Corpus Callosum | -0.008 | -0.318 | .751 |  | .469 |
| R Superior Longitudinal Fasciculus | 0.026 | 0.972 | .331 |  | .402 |
| L Superior Longitudinal Fasciculus | 0.029 | 1.104 | .270 |  | .453 |
| R Temporal Superior Longitudinal Fasciculus | -0.001 | -0.042 | .966 |  | .392 |
| L Temporal Superior Longitudinal Fasciculus | 0.025 | 0.956 | .339 |  | .443 |
| R Parietal Superior Longitudinal Fasciculus | 0.035 | 1.255 | .210 |  | .385 |
| L Parietal Superior Longitudinal Fasciculus | 0.028 | 1.064 | .288 |  | .443 |
| R Superior Corticostriate | 0.087 | 3.210 | .001 |  | .409 |
| L Superior Corticostriate | 0.068 | 2.612 | .009 |  | .442 |
| R Superior Corticostriate-frontal cortex only | 0.095 | 3.445 | .001 | * | .377 |
| L Superior Corticostriate-frontal cortex only | 0.057 | 2.195 | .028 |  | .456 |
| R Superior Corticostriate-parietal cortex only | 0.085 | 3.144 | .002 |  | .404 |
| L Superior Corticostriate-parietal cortex only | 0.081 | 3.000 | .003 |  | .416 |
| R Striatal Inferior Frontal Cortex | -0.027 | -1.177 | .239 |  | .564 |
| L Striatal Inferior Frontal Cortex | -0.019 | -0.804 | .422 |  | .561 |
| R Inferior Frontal Superior Frontal Cortex | 0.048 | 1.687 | .092 |  | .353 |
| L Inferior Frontal Superior Frontal Cortex | 0.006 | 0.206 | .837 |  | .368 |
| R Fornix, excluding fimbria | 0.011 | 0.341 | .733 |  | .110 |
| L Fornix, excluding fimbria | 0.032 | 0.996 | .319 |  | .138 |
| R Hemisphere Tract Fibers | 0.015 | 0.647 | .518 |  | .568 |
| L Hemisphere Tract Fibers | 0.012 | 0.500 | .617 |  | .552 |
| Notes: *p <.0013; All regressions control for age, age sq, sex, GAF, and scanner. | | | | | |

| Table II. Regression results of relations of family income to volume in each fiber tract (n = 1036). | | | | | |
| --- | --- | --- | --- | --- | --- |
|  | Beta | t | p-value |  | Adj R sq |
| R Fornix | -0.025 | -1.001 | .317 |  | .503 |
| L Fornix | -0.003 | -0.116 | .907 |  | .563 |
| R Cingulate Cingulum | 0.004 | 0.158 | .874 |  | .531 |
| L Cingulate Cingulum | 0.016 | 0.661 | .509 |  | .503 |
| R Parahippocampal Cingulum | 0.008 | 0.273 | .785 |  | .378 |
| L Parahippocampal Cingulum | -0.006 | -0.231 | .817 |  | .371 |
| R Corticospinal/Pyramidal | 0.028 | 1.714 | .087 |  | .783 |
| L Corticospinal/Pyramidal | 0.038 | 2.277 | .023 |  | .776 |
| R Anterior Thalamic Radiations | -0.006 | -0.310 | .757 |  | .741 |
| L Anterior Thalamic Radiations | -0.012 | -0.626 | .531 |  | .707 |
| R Uncinate | -0.022 | -1.051 | .294 |  | .662 |
| L Uncinate | -0.028 | -1.206 | .228 |  | .573 |
| R Inferior Longitudinal Fasciculus | 0.010 | 0.519 | .604 |  | .688 |
| L Inferior Longitudinal Fasciculus | 0.007 | 0.381 | .703 |  | .721 |
| R Inferior-Fronto-Occipital Fasciculus | 0.023 | 1.269 | .205 |  | .743 |
| L Inferior-Fronto-Occipital Fasciculus | 0.018 | 0.938 | .349 |  | .706 |
| Foreceps Major | 0.033 | 1.557 | .120 |  | .651 |
| Foreceps Minor | -0.041 | -2.078 | .038 |  | .689 |
| Corpus Callosum | 0.001 | 0.088 | .930 |  | .834 |
| R Superior Longitudinal Fasciculus | 0.029 | 1.536 | .125 |  | .710 |
| L Superior Longitudinal Fasciculus | -0.006 | -0.296 | .767 |  | .697 |
| R Temporal Superior Longitudinal Fasciculus | 0.031 | 1.482 | .139 |  | .658 |
| L Temporal Superior Longitudinal Fasciculus | 0.007 | 0.346 | .729 |  | .676 |
| R Parietal Superior Longitudinal Fasciculus | 0.010 | 0.582 | .561 |  | .742 |
| L Parietal Superior Longitudinal Fasciculus | <.001 | -0.004 | .996 |  | .743 |
| R Superior Corticostriate | 0.033 | 1.671 | .095 |  | .681 |
| L Superior Corticostriate | 0.020 | 1.129 | .259 |  | .760 |
| R Superior Corticostriate-frontal cortex only | 0.049 | 2.697 | .007 |  | .738 |
| L Superior Corticostriate-frontal cortex only | 0.015 | 0.858 | .391 |  | .765 |
| R Superior Corticostriate-parietal cortex only | 0.021 | 1.004 | .315 |  | .652 |
| L Superior Corticostriate-parietal cortex only | 0.031 | 1.521 | .129 |  | .678 |
| R Striatal Inferior Frontal Cortex | -0.016 | -0.784 | .433 |  | .667 |
| L Striatal Inferior Frontal Cortex | -0.014 | -0.603 | .547 |  | .571 |
| R Inferior Frontal Superior Frontal Cortex | 0.002 | 0.134 | .893 |  | .736 |
| L Inferior Frontal Superior Frontal Cortex | -0.042 | -2.327 | .020 |  | .735 |
| R Fornix, excluding fimbria | -0.035 | -1.212 | .226 |  | .349 |
| L Fornix, excluding fimbria | 0.010 | 0.401 | .689 |  | .459 |
| Right Hemisphere Tract Fibers | 0.004 | 0.362 | .718 |  | .886 |
| Left Hemisphere Tract Fibers | -0.003 | -0.231 | .817 |  | .885 |
| Notes: *p <.0013; All regressions control for age, sex, GAF, whole brain volume, and scanner. | | | | | |

| Table III. Regression results of relations of parental education to FA in each tract (n = 1047). | | | | | |
| --- | --- | --- | --- | --- | --- |
|  | Beta | t | p-value |  | Adj R sq |
| R Fornix | 0.053 | 1.656 | .098 |  | .141 |
| L Fornix | 0.045 | 1.373 | .170 |  | .127 |
| R Cingulate Cingulum | -0.003 | -0.125 | .901 |  | .503 |
| L Cingulate Cingulum | -0.030 | -1.199 | .231 |  | .499 |
| R Parahippocampal Cingulum | 0.029 | 0.977 | .329 |  | .295 |
| L Parahippocampal Cingulum | 0.089 | 2.985 | .003 |  | .262 |
| R Corticospinal/Pyramidal | 0.051 | 1.764 | .078 |  | .300 |
| L Corticospinal/Pyramidal | 0.069 | 2.321 | .020 |  | .264 |
| R Anterior Thalamic Radiations | 0.010 | 0.407 | .684 |  | .465 |
| L Anterior Thalamic Radiations | -0.008 | -0.306 | .760 |  | .406 |
| R Uncinate | 0.028 | 1.086 | .278 |  | .453 |
| L Uncinate | 0.024 | 0.804 | .422 |  | .288 |
| R Inferior Longitudinal Fasciculus | 0.056 | 2.353 | .019 |  | .528 |
| L Inferior Longitudinal Fasciculus | 0.053 | 2.253 | .024 |  | .552 |
| R Inferior-Fronto-Occipital Fasciculus | 0.044 | 1.924 | .055 |  | .577 |
| L Inferior-Fronto-Occipital Fasciculus | 0.050 | 2.185 | .029 |  | .572 |
| Foreceps Major | -0.003 | -0.100 | .920 |  | .407 |
| Foreceps Minor | 0.019 | 0.614 | .539 |  | .238 |
| Corpus Callosum | -0.002 | -0.077 | .938 |  | .464 |
| R Superior Longitudinal Fasciculus | 0.042 | 1.545 | .123 |  | .401 |
| L Superior Longitudinal Fasciculus | 0.050 | 1.940 | .053 |  | .447 |
| R Temporal Superior Longitudinal Fasciculus | 0.020 | 0.730 | .466 |  | .389 |
| L Temporal Superior Longitudinal Fasciculus | 0.047 | 1.805 | .071 |  | .437 |
| R Parietal Superior Longitudinal Fasciculus | 0.047 | 1.700 | .089 |  | .383 |
| L Parietal Superior Longitudinal Fasciculus | 0.041 | 1.574 | .116 |  | .439 |
| R Superior Corticostriate | 0.073 | 2.715 | .007 |  | .403 |
| L Superior Corticostriate | 0.073 | 2.803 | .005 |  | .449 |
| R Superior Corticostriate-frontal cortex only | 0.082 | 2.975 | .003 |  | .377 |
| L Superior Corticostriate-frontal cortex only | 0.053 | 2.089 | .037 |  | .468 |
| R Superior Corticostriate-parietal cortex only | 0.068 | 2.526 | .012 |  | .396 |
| L Superior Corticostriate-parietal cortex only | 0.088 | 3.293 | .001 | * | .414 |
| R Striatal Inferior Frontal Cortex | -0.001 | -0.061 | .951 |  | .565 |
| L Striatal Inferior Frontal Cortex | < .001 | -0.018 | .985 |  | .564 |
| R Inferior Frontal Superior Frontal Cortex | 0.025 | 0.891 | .373 |  | .357 |
| L Inferior Frontal Superior Frontal Cortex | 0.028 | 0.996 | .319 |  | .370 |
| R Fornix, excluding fimbria | 0.028 | 0.848 | .397 |  | .103 |
| L Fornix, excluding fimbria | 0.045 | 1.382 | .167 |  | .130 |
| R Hemisphere Tract Fibers | 0.028 | 1.226 | .221 |  | .565 |
| L Hemisphere Tract Fibers | 0.031 | 1.304 | .192 |  | .548 |
| Notes: *p <.0013; All regressions control for age, age sq, sex, GAF, and scanner. | | | | | |

| Table IV. Regression results of relations of parental education to volume in each fiber tract (n = 1047). | | | | | |
| --- | --- | --- | --- | --- | --- |
|  | Beta | t | p-value |  | Adj R sq |
| R Fornix | -0.015 | -0.587 | .557 |  | .500 |
| L Fornix | -0.014 | -0.595 | .552 |  | .554 |
| R Cingulate Cingulum | -0.018 | -0.761 | .447 |  | .528 |
| L Cingulate Cingulum | -0.014 | -0.575 | .565 |  | .498 |
| R Parahippocampal Cingulum | < .001 | < .001 | 1.000 |  | .370 |
| L Parahippocampal Cingulum | -0.011 | -0.406 | .685 |  | .362 |
| R Corticospinal/Pyramidal | -0.008 | -0.488 | .625 |  | .781 |
| L Corticospinal/Pyramidal | 0.010 | 0.626 | .531 |  | .780 |
| R Anterior Thalamic Radiations | -0.013 | -0.752 | .452 |  | .748 |
| L Anterior Thalamic Radiations | 0.007 | 0.373 | .709 |  | .718 |
| R Uncinate | -0.029 | -1.419 | .156 |  | .672 |
| L Uncinate | -0.008 | -0.340 | .734 |  | .582 |
| R Inferior Longitudinal Fasciculus | 0.035 | 1.809 | .071 |  | .689 |
| L Inferior Longitudinal Fasciculus | 0.024 | 1.307 | .192 |  | .718 |
| R Inferior-Fronto-Occipital Fasciculus | 0.020 | 1.134 | .257 |  | .740 |
| L Inferior-Fronto-Occipital Fasciculus | 0.014 | 0.734 | .463 |  | .707 |
| Foreceps Major | -0.014 | -0.687 | .492 |  | .646 |
| Foreceps Minor | -0.012 | -0.622 | .534 |  | .696 |
| Corpus Callosum | -0.022 | -1.514 | .130 |  | .833 |
| R Superior Longitudinal Fasciculus | -0.002 | -0.127 | .899 |  | .718 |
| L Superior Longitudinal Fasciculus | 0.012 | 0.608 | .543 |  | .701 |
| R Temporal Superior Longitudinal Fasciculus | -0.006 | -0.290 | .772 |  | .664 |
| L Temporal Superior Longitudinal Fasciculus | 0.026 | 1.300 | .194 |  | .679 |
| R Parietal Superior Longitudinal Fasciculus | -0.004 | -0.234 | .815 |  | .748 |
| L Parietal Superior Longitudinal Fasciculus | -0.002 | -0.114 | .910 |  | .747 |
| R Superior Corticostriate | 0.015 | 0.772 | .440 |  | .678 |
| L Superior Corticostriate | -0.004 | -0.250 | .803 |  | .761 |
| R Superior Corticostriate-frontal cortex only | <.001 | 0.020 | .984 |  | .737 |
| L Superior Corticostriate-frontal cortex only | -0.008 | -0.470 | .638 |  | .768 |
| R Superior Corticostriate-parietal cortex only | 0.018 | 0.871 | .384 |  | .650 |
| L Superior Corticostriate-parietal cortex only | 0.010 | 0.489 | .625 |  | .679 |
| R Striatal Inferior Frontal Cortex | -0.019 | -0.926 | .355 |  | .671 |
| L Striatal Inferior Frontal Cortex | -0.037 | -1.624 | .105 |  | .581 |
| R Inferior Frontal Superior Frontal Cortex | -0.049 | -2.758 | .006 |  | .747 |
| L Inferior Frontal Superior Frontal Cortex | -0.063 | -3.507 | < .001 | * | .742 |
| R Fornix, excluding fimbria | -0.040 | -1.407 | .160 |  | .349 |
| L Fornix, excluding fimbria | -0.031 | -1.205 | .228 |  | .450 |
| Right Hemisphere Tract Fibers | -0.017 | -1.439 | .151 |  | .885 |
| Left Hemisphere Tract Fibers | -0.014 | -1.175 | .240 |  | .885 |
| Notes: *p <.0013; All regressions control for age, sex, GAF, whole brain volume, and scanner. | | | | | |
